# An integrative omics approach to unravel toxicity mechanisms of environmental chemicals: effects of a formulated herbicide

**DOI:** 10.1038/s41598-018-29662-6

**Published:** 2018-07-27

**Authors:** Tiago Simões, Sara C. Novais, Tiago Natal-da-Luz, Bart Devreese, Tjalf de Boer, Dick Roelofs, José P. Sousa, Nico M. van Straalen, Marco F. L. Lemos

**Affiliations:** 1 MARE – Marine and Environmental Sciences Centre, ESTM, Polytechnic Institute of Leiria, Peniche, Portugal; 2 Centre for Functional Ecology, Department of Life Sciences, University of Coimbra, Coimbra, Portugal; 3 Institute of Ecological Sciences, Vrije University, Amsterdam, Netherlands; 4 Laboratory for Microbiology (LM-Ugent), Ghent University, Ghent, Belgium

## Abstract

The use of integrative molecular approaches can aid in a comprehensive understanding of the effects of toxicants at different levels of biological organization, also supporting risk assessment. The present study aims to unravel the toxicity mechanisms of a widely used herbicide to the arthropod *Folsomia candida* exposed in a natural soil, by linking effects on reproduction, proteomics and genome-wide gene expression. The EC50 effects on reproduction over 4 weeks was 4.63 mg glyphosate/kg of soil. The formulation included a polyethoxylated tallowamine as an adjuvant, which at 50% effect on reproduction had an estimated concentration of 0.87–1.49 mg/kg of soil. No effects were observed on survival and reproduction when using the isolated active substance, pointing the toxicity of the formulated product to the co-formulant instead of the active ingredient, glyphosate. RNA sequencing and shotgun proteomics were applied to assess differential transcript and protein expressions between exposed and control organisms in time, respectively. Specific functional categories at protein and transcriptome levels were concordant with each other, despite overall limited correlations between datasets. The exposure to this formulation affected normal cellular respiration and lipid metabolism, inducing oxidative stress and leading to impairment in biological life cycle mechanisms such as molting and reproduction.

## Introduction

Due to the high complexity of ecosystems, environmental risk assessment can be a very challenging task. There is a need to develop and validate innovative and reliable tools and integrated approaches for fast detection of changes in population and community structures that can be applied by regulatory agencies^1^. The application of omics tools (genomics, transcriptomics, proteomics, and metabolomics) to evaluate environmental health status is becoming increasingly important in environmental management and is helpful for risk assessment. Omics may allow connecting molecular events and adverse outcomes with biological levels of organization relevant for risk assessment schemes^2,3^. The analysis of the abundance of transcripts of RNA^4–7^ or more recently proteomics^8–11^ have already gained particular interest by helping to better understand the mechanistic interactions between the stressor and the organism at different stress conditions, but also to unravel the modes of action (MoA) of several toxicants.

Due to translational regulatory events, the difference in the average half-life of molecules, or even the physical properties of transcripts^12^, abundance of mRNA does not always correlate with protein abundance (e.g. Gygi *et al*.)^13^, particularly in complex biological samples^14^. However, the analysis of both datasets from the same experiment may provide useful insights that may not be deciphered from individual analysis of gene expression or protein levels.

Collembola (springtails) are a group with high abundance in almost any environment, and *Folsomia candida* has emerged as a standard species and genomic model organism for soil-toxicology^15^. Several studies have addressed the effects of different stressors, such as metals^16,17^, pesticides^18,19^ or combined pollutants^20^, in gene expression of this collembolan species over the last decade. Since then, considerable advances have been made to assemble a more complete transcriptome of *F*. *candida*^21^ and the complete genome for this species was also very recently made available^22^. However, proteomic information is still lacking. To our knowledge, there are no proteomic studies available for *F*. *candida* and the present study represents the first insight on its proteome.

In agricultural fields, pesticides are applied as complex commercial formulations, whose active ingredients have commonly relatively well characterized effects on target, as well and some non-target organisms. However, for some herbicide formulations, the added adjuvants confer higher or different routes of toxicity. It is therefore of major importance to also understand the general effects of the applied formulations.

Glyphosate-based herbicides are between the most used and studied formulations around the globe, with their future usage being currently under great debate by the European Union (EU). In a recent study^23^, 317 EU agricultural topsoils from eleven European countries were analyzed and glyphosate and/or its primary metabolite were detected in 45% of the tested samples, with a maximum concentration of 2 mg kg^−1^.

This work primarily intended to better understand the mechanisms of toxicity of a glyphosate-based formulation to the soil ecotoxicological model organism *F*. *candida*, by linking effects at different levels of biological organization: reproduction, protein levels, and gene expression. In addition, the potential of integrative gene and protein expression analysis as potential early warning indicators to be applied in environmental assessment studies was evaluated.

## Methods

### Test soil, organisms and contaminant

The test soil was collected from an agricultural area from the lower Mondego valley in Coimbra, Portugal (40°12′44.0″N; 8°27′02.4″W). The area was kept in fallow for over 10 years without use of pesticides. After collection, soil was sieved (5 mm), and defaunated using freezing-thawing cycles, being then kept at 4 °C for a maximum of 1 month. The physicochemical and mineralogical characterization of the natural soil is provided in Supplementary Table S1.

The collembolan *F*. *candida*, obtained from cultures of the Laboratory of Soil Ecology and Ecotoxicology of the Centre for Functional Ecology, University of Coimbra (Portugal), was used as test species. Cultures were maintained while kept on a wet mixture of plaster of Paris and activated charcoal (11:1, w/w), at 20 ± 2 °C and with a 16:8 h light:dark photoperiod, and fed baker’s yeast (Vahiné, Mccormick, Italy).

The herbicide formulation Montana® was acquired from SAPEC, Portugal, with a declared component composition including glyphosate (CAS 1071-83-6) as the active ingredient (30.8%, a.i.) in the form of isopropyl ammonium salt (C_6_H_17_N_2_O_5_P) and a polyethoxylated tallow amine adjuvant (POEA; CAS 61791-26-2) with concentrations ranging from 7 to 12% (w/w). After spiking the herbicide formulation into soil, glyphosate concentrations in soil samples were determined using LC-MS/MS by an external accredited company (Eurofins Scientific, Portugal), following methanol extraction. The storage time before analysis was approximately 1 month. The POEA adjuvants are very complex mixtures and their quantification in formulations is currently very challenging^24^. Therefore, the spiking calculations were based on the active ingredient (glyphosate) present in the formulation. We used the ratio between glyphosate and POEA in the product label to estimate POEA concentrations mixed into soil at specific effect levels.

### Reproduction tests and exposure for the omics analyses

To find the concentration causing 50% inhibitory effect on reproduction (EC50), several dilutions of the formulation were prepared in distilled water and tested after mixing them into soil portions, according to nominal concentrations of the active ingredient, to achieve the test concentrations of 0.135; 0.27; 0.54; 1.08; 2.16 and 4.32 mg a.i./kg of dry soil. For toxicity comparison purposes, a parallel reproduction test was conducted with pure glyphosate (Sigma-Aldrich, USA), testing the same concentrations as in the formulation (plus two additional concentrations: 8.64 and 17.28 mg a.i/kg). The reproduction tests were conducted according to ISO guideline 11267^25^ recommendations. Briefly, synchronized organisms (10–12 days old) were exposed to the above-mentioned concentrations, plus control, maintaining temperature and photoperiod at 20 ± 2 °C and 16:8 h light:dark. Five replicates per treatment were used, each with 10 organisms in 30 g of soil in a 100 mL glass vial. Organisms were fed after 14 days (2 mg of dry yeast spread in crumbs on the surface) and humidity was verified weekly by adding distilled water to the original weight. After 28 days of exposure, adults and juveniles present in each replicate were floated to the surface, a digital picture was taken and the animals were counted using image analysis software provided by ImageJ (version 1.49). The inhibitory effect on reproduction (EC50) was calculated by fitting a four-parameter logistic dose response curve (y = a + (b − a)/(1 + 10^(log(c)−x)*d)^) through the concentration response data, with “a” being the average juveniles at the highest concentration tested (bottom plateau), “b” the average juveniles at the control treatment (top plateau), “c” being the EC50 and “d” the hill’s slope. Fitting was done with Statistica software (version 7, Statsoft, 2004). Data homoscedasticity and normality was observed a posteriori via a residue analysis and a Q-Q plot. For evaluating survival and reproduction statistical effects, and depending on the fulfillment of the assumptions of normality and homoscedasticity, either a one-way analysis of variance (ANOVA) or a Kruskal-Wallis was used, respectively.

For the mechanistic assessment of the effects caused by the formulation, and to better understand the correlations between transcriptomics and proteomics information through time, organisms were then exposed to the EC50 (calculated through the reproduction test), using the formulation (plus control) and sampled at different time-points (4, 7, and 10 days). The exposure was performed with slight modifications from the reproduction tests: four replicates per treatment and time-point were used (24 in total), consisting of vials with 50 g of soil and 75 organisms. The organisms were fed only at the beginning of the exposure period with 2 mg of dry yeast. In parallel, to follow and verify the actual reproduction and mortality effects of the EC50 exposure after 28 days, 5 extra replicates from each treatment were used with 30 g of soil and 10 organisms, following the same ISO guideline recommendations^25^.

### Biological material extraction and isolation

The total number of organisms recovered from each test vial were pooled and considered a replicate for the omics analyses. After 4, 7 and 10 days of exposure, average organisms recovered per replicate were, respectively, 70.3 ± 7.6, 70.0 ± 7.5, 75.0 ± 0.0 for control samples and 72.0 ± 7.7, 73.3 ± 3.7, 70.2 ± 7.2 for contaminated samples. Both RNA and proteins were extracted from the same pools of animals, using the TRIZOL® extraction method (Invitrogen, Belgium). After the first RNA precipitation, wash and resuspension steps, total RNA was submitted to a DNase treatment for RNA purification and further resuspended in DEPC water (20 µL). RNA integrity was checked for every sample before proceeding with the preparation for RNA-sequencing. For this purpose, 500 ng from each sample were analysed with an Agilent RNA 6000 Nano Kit in a 2100 Bioanalyzer equipment (Agilent Technologies, USA). Total RNA was quantified using the Nanodrop 2000 (Thermo Scientific, USA). DNA contamination was also assessed with Qubit® 2.0 Fluorometer (ThermoFisher Scientific, USA), and concentrations of this nucleic acid in the RNA samples were generally lower than 10 ng/µL (less than 1% contamination). RNA samples were kept at −80 °C until further analysis.

Total proteins were precipitated and washed according to TRIZOL® protocol and resuspended in 200 µL of the following buffer: 40 mM Tris-HCl (pH 8.0) with 7 M urea, 2 M thiourea, 1% ASB-14, 0.5% triton-X-100, 0.1% SDS, 50 mM MgCl_2_, 0.5 mg/ml bovine pancreas DNase I (Roche, Germany), 0.25 mg/ml bovine pancreas RNase A (Roche, Germany), and a protease inhibitor cocktail mixture (Roche, Germany), according to the manufacturer recommendations. Total protein was quantified using Pierce™ Coomassie (Bradford) Protein Assay Kit (ThermoFisher Scientific, USA). After quantification, about 5 µg were used to run an electrophoretic SDS-Page gel for protein integrity and extraction efficiency determinations. Protein samples were kept at −80 °C until further analysis.

### Transcriptomics

About 1 µg of total RNA from each sample was used for mRNA selection and followed the TruSeq™ RNA Library Preparation Kit v2 (Illumina®) manufacturer’s protocol to be converted into a library of DNA fragments ready for sequencing. Briefly, mRNA was fragmented and converted to cDNA. After second strand synthesis, the steps included end repair, ligation of the sequencing adapters and the enrichment of 200–500 base pairs (bp) fragments. Quality and concentration of the fragmented and enriched library was verified with Agilent DNA 7500 Kit in the Agilent 2100 Bioanalyzer equipment (Agilent Technologies, USA). DNA libraries were normalized to 10 nM and pooled for sequencing. Next-generation sequencing (NGS) of the *F*. *candida* fragments was performed on the Illumina HiSeq2000 platform (Illumina, USA), following a Paired-End RNA Sequencing with 2 × 100 bp read length.

The resulting sequencing reads were mapped against the annotated transcriptome of *F*. *candida*^21^ using Bowtie software^26^, generating the total number of reads per transcript. Differentially expressed transcripts were obtained using Limma and edgeR packages in R (version 3.1.3), following a GLM (Generalized Linear Model) regression, with false discovery rate (FDR) correction for multiple testing. Transcripts with adjusted *p*-value below 0.05, after FDR correction, were considered to be differentially expressed. Gene Ontology (GO) enrichment analysis of the differentially expressed transcripts was performed using topGO package on R, using Fisher exact test with the weight function (p < 0.05). Clustering analysis of gene expression data was performed using MeV 4.9.0 software^27^, following the Self Organizing Tree Algorithm (SOTA), Pearson Uncentered metric distance, and cell diversity as cell division criteria. Gene interaction analysis was performed using GeneMANIA plugin from Cytoscape software (version 3.4.0)^28^.

### Proteomics

Protein denaturation, reduction, digestion with trypsin and labelling of the digested samples with the isobaric tags, were performed according to the iTRAQ® Reagents - 8 plex protocol (Applied Biosystems, USA). Total amount from each sample was normalized to exactly 20 µg and each sample to be directly compared was subsequently labeled with different isobaric tags. After being randomly labelled, samples to be directly compared were pooled together (8 samples for iTRAQ-8plex: 4 control and 4 pesticide EC50 treatment samples from the same time-point). Samples were dried in a SC110 Savant™ Speedvac (ThermoFisher Scientific, USA) and stored at −20 °C until analysis. Afterwards, samples underwent a 2 dimension reversed phase (RP) high-pressure liquid chromatographic (HPLC) separation of the peptides: an alkaline fractionation (pH = 10), followed by acidic fractionation (pH = 3). Conditions for both fractionations can be consulted in Supplementary Text. The fractions of interest were collected, pooled into six different fractions and conditioned for the next separation by drying in the Speedvac, followed by resuspension in 20 µl of 50% ACN with 0.1% trifluoroacetic acid (TFA) and stored at −20 °C. Of these fractions, 10 µL was loaded for the acidic fractionation. The effluent was directly spotted by the Probot device (Dionex-LC Packings) on an OptiTOF™ LC/MALDI target plate (512 spots), with 30 s intervals. The plates were previously spotted with the supporting matrix, consisting of 4 mg/mL α-cyano-4-hydroxycinnamic acid, 70% ACN, 0.1% TFA, 10 mM dibasic ammonium citrate and 0.01 pmol/µL Glu-fibrinopeptide B. A 4800 Plus MALDI-TOF/TOF analyzer mass spectrometer (AB SCIEX, USA) was used for the analysis. Identification of the peptides was based on both MS and MS/MS information using the combined peaks for all iTRAQ® labelled peptides, whereas the relative quantitation of the peptides was accomplished in the MS/MS mode using the peak intensity of the eight different iTRAQ® reporter ions. All spectra were analyzed and compiled with 4000 Series Explorer™ Software. ProteinPilot™ software (version 4.0), involving the Paragon Pro-Group algorithms from ABSciex^29^, was used for identification and relative quantitation of peptides and correspondent predicted proteins, using *F*. *candida* genome as database^22^. The selected parameters were: iTRAQ-label modification at the peptide level, fixed MMTS modification of cysteine, variable biological modifications and amino acid substitutions. Background correction was applied. A false discovery rate (FDR) analysis was performed automatically by the software. After, the proteins with a local FDR <5% were exclusively retained. Of these, only proteins with an “unused score” greater than 1.0 (identification with 90% confidence) and quantified in all replicates, were considered for further analysis. Furthermore, to address differences between control and treatment samples, a two-tailed Student’s *t*-test was performed on the logarithmic transformed ratios and the significance level was set at *p* < 0.05.

### Data availability

The datasets generated and analyzed during the current study were made available as Supplementary Datasets S4 (differentially expressed transcripts list), S5 (Functional annotation), S6 (detected protein levels). Raw Illumina sequence reads were deposited to the NCBI SRA under accession SRP115951 (Bioproject PRJNA399149).

## Results

### Effects on survival and reproduction

The measured concentrations of glyphosate in the soil samples showed an average deviation of 17% (<10% in the upper concentrations) compared to the nominal concentrations (see Supplementary Table S2). The estimated effect concentrations were based on the analytical concentrations. The reproduction tests with formulation and pure substance fulfilled the validity criteria described by the standard guideline^25^. No effects on survival were observed in the treatments (*F*_6,28_ = 0.916, *p* = 0.498). The estimated EC50 of the formulation on reproduction was 4.63 mg a.i./kg (95% CI: 0.58–8.68 mg/kg). As for the results with the active ingredient alone, no effects on *F*. *candida* survival nor on reproduction were observed (*H* = 6.257, df = 7, p = 0.510 and *F*_7,32_ = 1.561, *p* = 0.183, respectively).

For the mechanistic assessment of the effects caused by the formulation, extra replicates were used to assess if the exposure to EC50 reduced reproduction significantly in this particular experiment (analytical concentration: 4.71 mg a.i./kg; estimated POEA concentration: between 0.87 and 1.49 mg/kg; Supplementary Table S2). After 28 days, on average 9.2 ± 0.45 adult organisms and 522.6 ± 117.13 juveniles were present in control replicates *versus* 9.0 ± 1.22 adults and 390.6 ± 51.77 juveniles present in EC50 concentration samples, producing no effects on survival (*t* = 0.34, df = 4, *p* = 0.37, one-tailed), but a significant reduction of approximately 25% in the reproductive output (t = 2.3, df = 4, *p* = 0.02, one-tailed).

### Transcriptomics

Illumina sequencing yielded 24.124.447 paired-end reads, after trimming. An average of 83.26 ± 3.46% of reads per sample was successfully mapped to the transcriptome (38.015 transcripts). Sequencing related statistics per sample can be consulted in Supplementary Table S3. Statistical analysis of differential expression resulted in a total of 117 transcripts differentially expressed over the three different exposure time-points to the formulation, with 8 transcripts differentially regulated after 4 days (8 up, 0 down), 7 differentially regulated after 7 days (7 up, 0 down) and 102 differentially regulated after 10 days (6 up and 96 down), evidencing no overlaps between gene lists from the different time-points. A gene cluster analysis on the complete list of differentially expressed transcripts can be consulted in Supplementary Fig. S1.

The 4-days exposure resulted in six affected annotated transcripts, three of which encoded for a calcium-activated chloride channel regulator. The 7 and 10 days exposures resulted in 4 and 59 affected transcripts with known homologies to functional proteins, most of which are related to lipid metabolism, body development, chitin metabolism, and reproduction. The list of all differentially expressed transcripts with significant blast homologies and correspondent fold change values can be found in Supplementary Table S4.

A GO enrichment analysis was performed individually for each time-point to statistically evaluate the likelihood of certain biological processes (BP) and molecular functions (MF) to be significantly enriched along time (Table 1).

**Table 1:** GO enrichment analysis of the differentially expressed transcripts after 4, 7 and 10 days of *F. candida* exposure to the herbicide formulation at the EC50 concentration.

GO ID	Domain	Term	Annotated	4 Days		7 Days		10 Days	
				Signif.	Adj. *p*-value	Signif.	Adj. *p*-value	Signif.	Adj. *p*-value
***Up*** **-** ***regulated***									
GO:0055114	BP	Oxidation-Reduction Process	1100	1	0.316	2	**0.0391**	0	1
GO:0016491	MF	Oxidoreductase Activity	1271	1	1	2	**0.0337**	0	1
***Down*** **-** ***regulated***									
GO:0006030	BP	Chitin Metabolic Process	155	0	1	0	1	4	**6.90E-06**
GO:0006508	BP	Proteolysis	827	1	0.245	0	1	11	**2.10E-05**
GO:0030704	BP	Vitelline Membrane Formation	19	0	1	0	1	2	**0.0016**
GO:0008654	BP	Phospholipid Biosynthetic Process	73	0	1	0	1	2	**0.00217**
GO:0008061	MF	Chitin binding	135	0	1	0	1	5	**2.70E-05**
GO:0003841	MF	1-acylglycerol-3-phosphate O-acyltransferase	6	0	1	0	1	2	**0.0001**
GO:0016787	MF	Hydrolase	2751	2	1	1	1	24	**0.00018**
GO:0004568	MF	Chitinase	27	0	1	0	1	2	**0.0024**
GO:0004181	MF	Metallocarboxypeptidase	30	0	1	0	1	2	**0.0029**

GOs presenting singletons were not considered. Significantly enriched GO terms at each time-point (Adjusted p-value < 0.05) are highlighted in bold. BP (Biological Process); MF (Molecular Functions); Signif. (Significant).

In general, the BP and MF categories significantly associated with up-regulated transcripts were related to oxidation-reduction processes (Table 1). Four BP and five MF were associated with down-regulated transcripts, involved in lipid and chitin metabolism, body development and reproduction (Table 1). All significant differential transcripts within each GO term are given in Supplementary Table S5.

### Proteomics

Protein profiling yielded a total of 101 potential proteins for which 89 (88.1%) known functional homologies were retrieved. The complete protein list can be consulted in Supplementary Table S6. From these proteins, 17 (16.8%) were found to be significantly different between control and contaminated samples, with twelve proteins being more abundant (seven after 7 days and five after 10 days) and five proteins less abundant (one after 7 days and four after 10 days) (Table 2).

**Table 2:** List of proteins with significant altered levels (p < 0.05) in *F. candida* after 4, 7 and 10 days of exposure to the herbicide formulation at the EC50 concentration.

Transcript Acc. Number	Protein Homolog	Unused Score	Log_2_ Fold Change	*p*-value
**7 Days**				
GAMN01010555	50S ribosomal protein L9	2.12	3.76	0.005
GAMN01032080	Cytochrome C	2	3.33	1.55E-06
GAMN01009208	Vitellogenin	8.07	2.71	0.001
GAMN01000325	Ribosomal Prot. S19 40S	2	1.84	0.008
GAMN01003635	Hsp 70 kDA cognate 2	1.62	1.79	0.012
GAMN01002103	ADP/ATP carrier protein	2	1.55	0.008
GAMN01006818	Vitellogenin-2	9.09	1.47	0.006
GAMN01009199	Cuticle protein 6	2	−0.64	0.029
**10 Days**				
GAMN01000323	Superoxide dismutase [Cu-Zn]	2	6.333	2.02E-06
GAMN01009208	Vitellogenin	14.68	3.97	0.017
GAMN01006818	Vitellogenin-2	14.63	3.03	0.022
GAMN01001328	Alpha-actinin, sarcomeric	2.56	1.46	0.002
GAMN01000430	Fatty acid-binding protein	3.77	0.591	0.003
GAMN01003161	Tropomyosin	14.35	−0.99	0.028
GAMN01001827	Catalase	2	−0.704	0.025
GAMN01004453	NDUFV2, mitochondrial	2	−0.64	0.014
GAMN01006823	Glutathione S-transferase 5	2	−0.591	0.049

The major biological functions of significantly altered proteins are involved in oxidative stress (e.g. superoxide dismutase, catalase, cytochrome c), response to xenobiotic compounds (glutathione S-transferase), and reproduction (e.g. vitellogenin, vitellogenin 2). Less representative processes affected are mitochondrial transport (ADP/ATP carrier protein and NDUFV2) and body development (cuticle protein 6).

After 4 days of exposure, there were no significant differences in protein abundances (see Supplementary Table S6) and a cuticle-related protein was the only significantly low abundant peptide after 7 days. In contrast, proteins such as cytochrome C, heat shock 70 kDa protein cognate 4, ADP-ATP carrier protein, and ribosomal proteins were significantly more abundant at exposure day 7. Egg yolk precursors such as vitellogenin and vitellogenin 2 were observed to become more abundant with time, with increasing values from 7 to 10 days in the contaminated samples.

The levels of detoxification and antioxidant related enzymes decreased after 10 days of exposure (e.g. glutathione S-transferase, catalase), whereas the antioxidant superoxide dismutase [Cu-Zn] was significantly higher, being up to 80 times more abundant comparatively to control samples.

### Gene and protein correlations

The whole set of identified proteins, and corresponding coding transcripts, were used for correlation analyses to increase the power of analysis (Pearson correlation coefficient, *r*_p_; *p* < 0.05). Several correlations were tested between datasets belonging to the same time-point, but also between time-points. The correlation coefficients and *p*-value results for all analyses are presented in Supplementary Table S7. Transcription levels of stress-related proteins at day 4 were positively correlated with their protein levels at day 10 (*r*_p_ = 0.60, *p* = 0.007), and this correlation increased when transcript levels at 10 days were compared to protein levels from the same time-point (*r*_p_ = 0.84, *p* = 0.002). Moreover, proteins related to motor activity were also significantly correlated between transcript and protein level at 4 days (*r*_p_ = 0.998, *p* = 0.011), but also between transcript levels at 4 days and protein levels at 7 days (*r*_p_ = 0.997, *p* = 0.047).

**Figure 1:**
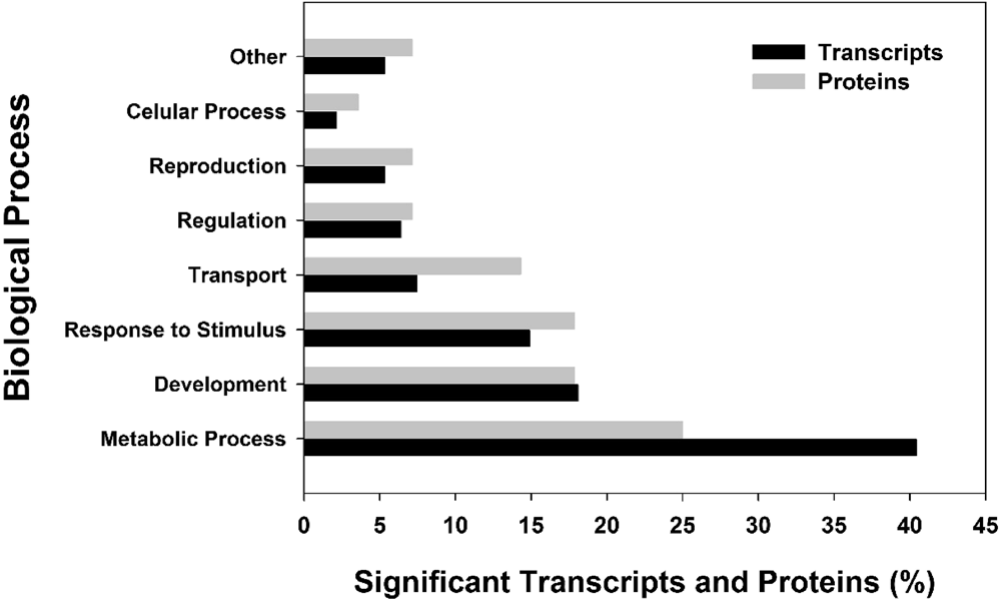
Categorization of all differential *F. candida* gene expression and protein levels classified by GO-term (Biological Process) after exposure to the herbicide formulation during 4, 7 and 10 days. Results are expressed in percentage (%). Blast2GO software (version 4.0) and UniProt database (www.uniprot.org) were used to group both differential transcript expression and protein levels into the GO categories, according to the major biological processes involved.

Despite the low significance of correlations between transcript and protein levels, there was substantial similarity in the biological processes drawn from associated GO terms (Fig. 1).

## Discussion

The exposure of *F*. *candida* to Montana®, a widely used herbicide formulation in Portugal resulted in a reproduction EC50 of approximately 5 mg a.i./kg of soil [95% CI: 0.58–8.68], when expressed in terms of the active ingredient glyphosate. At this concentration of active ingredient, the adjuvant concentration in soil is estimated between 0.87–1.49 mg POEA/kg soil.

It is doubtful whether the effects of Montana® on *F*. *candida* are due to glyphosate. No effects were observed on either reproduction or survival when the pure active ingredient was tested, even at concentrations higher than in the tested formulation. According to Madsen *et al*.^30^, glyphosate was initially patented as a chelator agent (U.S. Patent No. 3.160.632, 1964) due to its propensity to bind metal cations, making it a strong protonophore^31^. It is therefore possible that when mixed into soil, the active ingredient becomes inactivated by binding to clay minerals, causing no effects in organisms at ecologically relevant concentrations, as was verified by Von Mérey *et al*.^32^ for earthworms, springtails, and predatory soil mites, and also in the present study for springtails. The differences between observations with the active substance and the formulation tested in our study must therefore consider the adjuvants used in the formulation to enhance stability and penetration of the active compounds into plant cells^33^. Adjuvants are declared as inert diluents, not being directly responsible for the intended pesticide activity^24^, although effects on other non-target organisms are known. According to the safety data sheet of the formulation tested in the present study, POEA was used as adjuvant, though the exact concentration was not mentioned (range from 7 to 12% p/p). Ethoxylated adjuvants are substances with a surfactant action, having alkyl chains and/or ethoxy groups in the ethoxylated chains, which can penetrate cell membranes disrupting their structure and functions^34^. Contrary to what is expected from a pesticide adjuvant, POEA has been reported in the last few years to present even higher toxicity to non-plant organisms than glyphosate alone^33,35^. A statement released by EFSA^36^ on this subject concluded that “compared to glyphosate, a higher toxicity of the POE-tallowamine was observed on all endpoints investigated”. This was the basis for the recommendation made by the EC in June 2016 (MEMO-16-2012) to ban POEA from glyphosate-based products, which has already been followed by some member states, including Portugal (the formulation used in this study was purchased prior to this market withdrawal). However, this adjuvant is still largely used worldwide not only in glyphosate-based formulations^24^, which increases the need to understand the real effects and the toxicity mechanisms of such formulations on non-target soil organisms of agricultural fields. Acute effects of POEA on the aquatic environment have already been largely addressed by Tush *et al*.^37^, where a list of acute toxic levels was presented for 24 aquatic species, or more recently in the bivalve mollusk *Crassostrea gigas*^38^. There is, however, limited information available for the terrestrial environment.

In this study, different responses to the formulation could be observed over time, at both gene and protein levels, highlighting the importance of following different time-points for a more accurate assessment of toxicity mechanisms. This pattern of temporal effects in gene expression profiles after exposure to chemical contaminants has been observed in previous ecotoxicogenomics studies with *F*. *candida* and other soil invertebrates^20,39–41^. Novais *et al*.^40^ exposed a potworm to the reproduction EC10 and EC50 of another herbicide and found a higher alteration in gene expression at day 2, with a subsequent decrease in the number of affected genes at day 4 and a late new increase in response after 21 days. This lead us to hypothesize that the lack of differential gene expression responses at 4 and 7 days in the present work, along with the observed increase in affected protein levels already at 7 days of exposure, might be a result of an earlier and undetected gene expression response (before 4 days of exposure), as it is often observed for early stress response enzymes such as catalase or glutathione S-transferase.

Nevertheless, the obtained results give valuable information about the mechanisms of toxicity of an herbicide formulation in this collembolan species, pointing towards induction of cellular oxidative stress, alterations in lipid metabolism, and impairment of development and reproduction. These mechanisms will be hereafter discussed in different sections, focusing on the integrative analysis of effects on both gene expression and protein levels.

### Cellular stress effects

Although major effects/responses to the formulation were only observed after 10 days, one of the first measured responses of *F*. *candida* to this exposure was the down-regulation of three transcripts (GAMN01012505, GAMN01007081, GAMN01014780) homologous to the calcium-activated chloride channel regulator 1 gene (CLCA1) at 4 days (Supplementary Fig. S1 and Supplementary Table S4). This gene encodes for a member of the calcium sensitive chloride conductance protein family, which is activated by calcium cations (Ca^2+^)^42^. There are studies with similar formulations to the one used here reporting cellular calcium disturbance upon exposure. In a work by De Liz Oliveira Cavalli *et al*.^43^ with Wistar rat testis or Sertoli cells exposed to another glyphosate-based formulation (Roundup®), the authors reported a Ca^2+^ imbalance and proposed that the formulation promotes an intracellular Ca^2+^ overload and subsequent cell signaling misregulation, potentially causing cellular stress. More recently, these Ca^2+^ imbalance effects were also discussed in a study addressing effects of glyphosate in marine invertebrate *Mytilus galloprovincialis*^44^. After 7 days of exposure, the response of the organisms to a stressful condition was evidenced by over-representation of transcripts involved in oxidation-reduction processes (GO: GO:0055114; Table 1). Two transcripts coding for a Tbh-like monooxygenase protein (Tbh, GAMN01003982, GAMN01004068; Supplementary Fig. S1), were significantly overexpressed, along with a glucosylceramidase homolog (GAMN01005039; CG33090) (Supplementary Fig. S1 and Supplementary Table S4). Arthropods synthesize tyramine beta-hydroxylase (TBH) instead of dopamine-β-hydroxylase (DBH), which converts tyramine into octopamine, the analogous neurotransmitter to noradrenaline of arthropods^45,46^. A stress response in insects is elicited by release of octopamine to cope with stressful energy demanding processes (e.g. flight response, locomotion)^45,47,48^. It is therefore expected that the overexpression of the gene encoding octopamine synthesis after 7 days, represents a response to stress induced by the contaminant. Glucosylceramidase (EC 3.2.1.45) is an enzyme involved in formation of D-glucose and sphingosines, which are important membrane signaling molecules. They are also commonly considered to protect cell surface against harmful environmental factors by forming a mechanically stable and chemically resistant outer leaflet of the plasma membrane lipid bilayer^49^. Thus, the up-regulation of these glucosylceramidase transcripts could indicate a strategy for springtails to protect their cells and avoid penetration of the xenobiotic metabolites.

Down-regulation of two collagen (Col14a1) homologs (GAMN01019740; GAMN01005839) was also observed after 10 days, which may be attributed to an increase in oxidative stress, as demonstrated by Webster *et al*.^50^ when exposing brown trout to formulated glyphosate (Roundup®). In addition to gene expression responses, stress related proteins such as Cyt C and heat shock 70 kDa protein cognate 4 increased. After 10 days, however, this tendency seemed to be reverted given that some stress response mechanisms were shown to be down-regulated (GO:0004181; catalase, NDUFV2; Tables 2 and S5). Nevertheless, an important antioxidant enzyme, a SOD homolog protein, was found to be highly abundant at 10 days. This is indicative of ROS accumulation, and specially superoxide anions. The high abundance of SOD suggests an extra need for antioxidants to cope with such radicals and cellular stress. Contardo-Jara *et al*.^51^ found similar effects in SOD activity when exposing the worm *Lumbriculus variegatus* to environmental concentrations in a Roundup® formulation during four days.

### Developmental and reproductive effects

**Figure 2:**
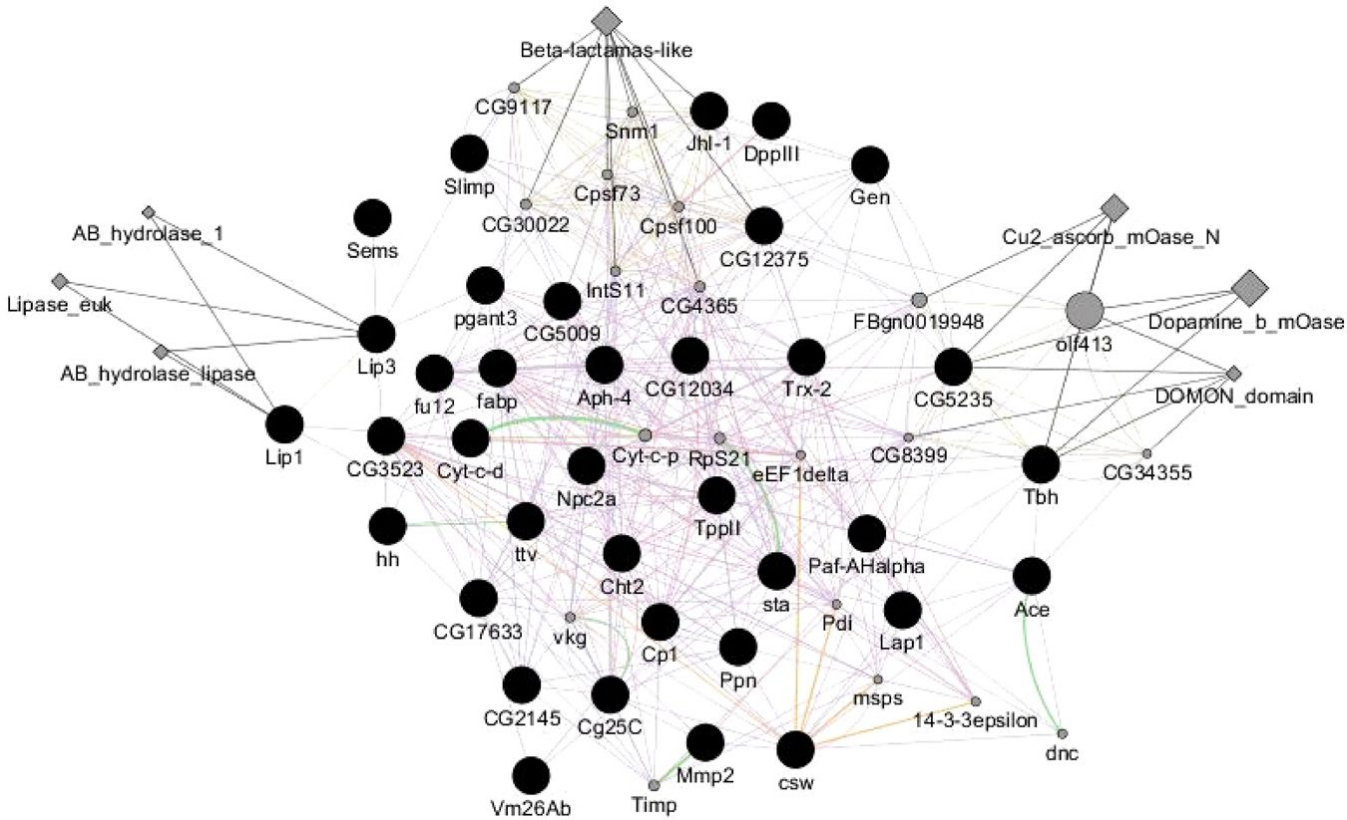
Gene interaction analysis [co-expression (purple), co-localization (light blue), physical interactions (orange), genetic interactions (green) and consolidated pathways (grey)] performed with *F*. *candida*’s differentially expressed transcripts after exposure to the herbicide formulation for 10 days. Protein domain attributes and genes related to query genes are presented in grey nodes and query genes presented in black nodes. Interaction analysis was performed with the list of *Drosophila melanogaster* genome homologous genes to *F*. *candida*, using GeneMANIA plugin from Cytoscape software (version 3.4.0).

The exposure to the herbicide formulation triggered different genes related to disturbances in lipid metabolism, namely by inhibiting the expression of transcripts involved in fatty acid elongation, sphingolipid metabolism, and steroid biosynthesis (for a KEGG^52^ pathway analysis see Supplementary Fig. S2). As lipids are key structural components of cellular membranes and participate in signaling and intracellular transport processes, they are expected to be among the first metabolic response agents to a stressor. There is evidence showing effects of glyphosate formulations on lipid metabolism in other non-target species through ROS production, steroid biosynthesis and ultimately, developmental and reproductive effects^38,50,53,54^. To note that after this exposure period the organisms were already 20–22 days old. Since *F*. *candida* reaches complete sexual maturity after 21 to 24 days of age^55^, this is indicative that at this stage the organisms most likely had started to reproduce. Several metabolic functions implied in body development (GO:0006030; GO:0006508; GO:0004568) and reproduction (GO:0030704) were down-regulated or inhibited after 10 days (Table 1). Molecular events related to reproduction are of particular interest, since they allow a direct link to the observed effect at a higher level of organization (25% inhibition on reproduction). The particular set of down-regulated genes after 10 days of exposure are presented in Fig. 2, illustrating physical and genetic interactions, co-expressions, and protein domain attributes.

A study in *Drosophila melanogaster* showed that 1-acylglycerol-3-phosphate-*O*-acyltransferase 2 (GAMN01037272, GAMN01010825; fu12) is mostly present in adipocytes and predicted to be involved in lipid storage and lipid droplet turnover during developmental stages and oogenesis^56^. Down-regulation of fu12 causes impairment of lipid mobilization, ultimately leading to developmental arrest and eventual embryo mortality, eventually unraveling the decreased reproductive rates verified in this work. The human homolog (AGPAT2) also plays a critical role in the early stages of growth and development of adipocytes, and in the biosynthesis of major components of cell membrane (glycerophospholipids)^57^, a process where the fatty acid synthase (GAMN01010449; CG3523) also participates. Fatty acid synthase is a multi-enzyme protein, catalyzing the formation of long-chain fatty acids from acetyl-CoA and malonyl-CoA, being expressed by lipogenic tissues, hormone-sensitive cells and proliferating fetal cells. Both genes have been previously shown to be implicated in adipocyte disturbance when down-regulated under xenobiotic contamination^58^. According to Fig. 2, these two genes are co-expressed under xenobiotic exposure^59^, which also seems to be applicable to the formulation tested here.

These mechanisms might also involve the observed expression inhibition of other co-expressed lipid related genes, such as the homolog to the human PAFAH1B1 (Paf-AHalpha; GAMN01013576), a putative sphingomyelinase (CG12034; GAMN1008482) and epididymal secretory protein homolog (Npc2a; GAMN01006832) (Fig. 2). The first two are involved in development and embryogenesis^60,61^. Npc2a could also be related to the developmental and reproduction effects since it plays an important role in cholesterol homeostasis and ultimate transport to plasma membrane, controlling the availability of sterol substrate and affecting steroidogenesis such as ecdysteroids^62^. Webster *et al*.^50^ also reported disturbances in cholesterol metabolism in fish after exposure to an herbicide formulation containing POEA.

The proteomics analysis also showed that vitellogenin (VTG) and vitellogenin-2 (VTG2) were significantly increased after 7 and 10 days of exposure (Table 2). In oviparous vertebrates and invertebrates, the egg yolk-precursor proteins are very important phospholipoglycoproteins involved in oocyte maturation^63^. Vitellogenin is produced in fat bodies and taken up by the developing oocytes through a specific receptor-mediated endocytosis in the oocyte outer membrane. After uptake into oocytes, the VTGs are stored in a crystalline form as vitellin, a reserve food-source for the future embryo development^64^. Although no up-regulation of vitellogenin genes (vtgs) was observed at any time-point, the accumulation of these proteins in exposed *F*. *candida* may be indicative that either their production was stimulated in fat cells/adipocytes or their conversion inside the oocyte was obstructed by some other mechanisms, revealing a clear disturbance in the reproductive cycle and embryogenesis that started to be noticed after 7 days of exposure. Provost-Javier and Rasgon^65^ reported a post-transcriptional regulation of vtgs, although using a different arthropod (mosquito). The authors demonstrated that the hormone 20-hydroxyecdysone (ecdysteroid hormone, 20E) was responsible for regulating post-transcriptional splicing of vtg through RUST (Regulated Unproductive Splicing and Translation) mechanism.

Oocyte maturation, fertilization, and embryo development are also general calcium mediated processes^63^. Webster *et al*.^50^ showed that low concentrations of Roundup® significantly affected calcium signaling pathways and calcium homeostasis in brown trout, disturbing reproduction. It cannot be excluded here that the reproductive impairment could also be a secondary effect caused by calcium imbalance induced by the formulation. Considering that calcium was described to be a good indicator of vitellogenin status in crustaceans and fish species^66^ and implied in vitellogenin synthesis^67^, it is therefore plausible the influence of calcium imbalance in the VTG accumulation observed for exposed organisms. In future research, it would be interesting to analyze the vitellin accumulation in the embryos of *F*. *candida* to further clarify the reproduction impairment mechanism involving these proteins. In a study with *Daphnia magna*, glyphosate did not induce alterations in vtg gene expression^68^, whereas when other authors tested Roundup® formulation, a significant effect in fecundity (embryogenesis) was observed^69^. The same reproductive effects were reported by Druart *et al*.^70^, exposing the terrestrial snail *Cantareus asperses* to an environmental concentration of a glyphosate-based herbicide containing 10 to 20% POEA (Bypass®). These reports raise some evidences that the reproductive effects observed in *F*. *candida* might be attributed to the surfactants of the formulation or possible synergistic effects and not to glyphosate itself.

There are also some indications that the impairment of reproduction could be related with a deficiency in octopamine hormone. The two transcripts involved in octopamine synthesis (tbh, mentioned in section 4.1), were found up-regulated at 7 days as a response to stress but were subsequently down-regulated at 10 days (Fig. 2, Supplementary Fig. S1 and Supplementary Table S4). Some studies with *D*. *melanogaster* and other insects have reported that the absence of TBH protein and consequent impairment of octopamine biosynthesis results in decreased egg deposition by a blockage of mature oocytes within the ovaries, given that octopamine is believed to modulate ovarian contractions and oviductal muscles for oviposition^47,71,72^. This Tbh gene appears to be co-expressed with papilin homolog gene in *D*. *melanogaster* (Ppn, GAMN01037019; Fig. 2), which is an essential extracellular matrix glycoprotein required for morphogenesis and hypodermal enclosure in the embryo^73,74^.

Downregulation of chitinase (Cht2, GAMN01037556; GAMN01007170) and transferase pgant 3 (GAMN01001593) are examples of developmental inhibition (Figs 2, S1 and Supplementary Tables S4 and S5). Cuticle proteins 6 and 7 were also detected in lower amounts in contaminated samples, although only cuticle protein 6 was found to be statistically decreased (Tables 2 and S6). In arthropods, cuticle proteins are constituents of the organism’s exoskeleton and together with chitinases they are key molecules in consecutive ecdysis and growth stages. The lower abundance of such proteins observed here suggests a slowdown in the development of *F*. *candida* when compared to control organisms. Similar results were obtained when Qiao *et al*.^75^ exposed the collembolan *F*. *candida* to the pesticide pentachlorophenol, where down-regulation of transcripts involved in molting processes was observed. The delay in development after contaminant exposure is a very well reported behavioral feature of molting organisms, including *F*. *candida*^76,77^.

### Gene and protein correlations

Generally for eukaryotes, the cellular concentrations of proteins and their corresponding RNAs have correlation coefficients of approximately 40%. The remaining 60% have been suggested to be mostly controlled at the translation level^78–80^. However, reports of higher rates can be found, especially in simpler biological systems such as bacteria and cell lines^81,82^, or when focusing on specific tissues^83^ and differentially expressed genes and proteins^78^. There are several biological aspects to consider when facing these poor correlations between datasets, such as transcriptional and translational machineries and regulatory events, which can also be tissue specific^84,85^ and can therefore result in different correlations for the same set of genes and proteins in different tissues^81^. Considering this and the fact that in the present study, the entire body composition of a pool of approximately 75 organisms was used in each replicate, it is possible that different organs and tissues could strongly contribute to the low correlations observed (see Supplementary Table S7).

Contrary to expectations, correlations between datasets from different time-points proved to be lower than those generated at identical time-points. The higher and most significant correlations between gene expression and protein levels were observed at 10 days for a specific subset of stress responses, which can also result from a higher number of stress regulation related genes and enzymes affected by the treatment at this time-point. Also important to mention is the fact that within the same time-point, RNA and proteins to be compared were extracted from exactly the same organisms and these higher correlations could be reflecting the lower inherent biological variation in these cases.

Additionally, only moderate protein diversity was identified throughout this study. There are several environmental proteomics reports indicating low amounts of proteins, possibly related to the biological complexity of the organisms or the proteome range methodologies available^83^, resulting in low correlations between RNA and protein levels. Many ecotoxicological reports refer to around 100 or less proteins identified in chitin based invertebrate species^86–89^ but also in other invertebrates^90,91^. Despite the low correlation rates observed here, the information provided by these two levels of datasets revealed to be complementary and corroborative.

## Conclusions

This study was based on state of the art methodologies (omics) to perform an integrated analysis for unraveling the mechanisms of toxic action of an herbicide formulation in *F*. *candida*. Despite the effects of the formulation, survival and reproduction were not affected by the active ingredient in the present study, even at higher concentrations. Omics analyses allowed identification of mechanisms of toxicity of the formulation, mainly through change in normal cellular respiration and lipid metabolism, leading to increased oxidative stress and impairment of molting and reproduction. These results presented higher similarities with other studies using analogous formulations or even with POEAs, inferred by an integrative analysis of transcript expression and protein abundance. Integrated omics could thus provide useful insights that could hardly be inferred from individual analysis of gene or protein expressions, proving the relevance of integrative analyses in toxicological studies.

## Electronic supplementary material

Supplementary Dataset 1
