# Supplementary material for: An integrative omics approach to unravel toxicity mechanisms of environmental chemicals: effects of a formulated herbicide
Source: Sci Rep. 2018 Jul 27;8:11376. doi: 10.1038/s41598-018-29662-6 (PMC6063884; doi:10.1038/s41598-018-29662-6)
Supplement: Supplementary file 1 — Supplementary Dataset 1 [file 41598_2018_29662_MOESM1_ESM.pdf]

Manuscript Title:

An integrative omics approach to unravel toxicity mechanisms of environmental chemicals: effects of a formulated herbicide.

Authors:

Tiago Simões[1,2,3*]; Sara C. Novais[1,3]; Tiago Natal-da-Luz[2]; Bart Devreese[4]; Tjalf de Boer[3]; Dick Roelofs[3]; José P. Sousa[2]; Nico Van Straalen[3]; Marco F.L. Lemos[1]

[1] MARE – Marine and Environmental Sciences Centre, ESTM, Polytechnic Institute of Leiria, Peniche, Portugal;

[2] Centre for Functional Ecology, Department of Life Sciences, University of Coimbra, Portugal;

[3] Institute of Ecological Sciences, Vrije University, Amsterdam, Netherlands;

[4] Unit for Structural Biology, Laboratory for Protein Biochemistry and Biomolecular Engineering (L-ProBE), Ghent University, Belgium;

Supplementary Table S1: Physicochemical and mineralogical characterization of the agricultural soil from the lower Mondego valley in Coimbra, Portugal.

Parameter	LOQ	Value	Unit
Determination from the original sample			
Dry substance (105ºC)	0.1	94.2	% w/w
Dry substance (105ºC)	0.1	94.2	% w/w OS
pH Value		7.1	
Loss of ignition of the dry substance	0.1	5.0	% w/w DS
Total Nitrogen	0.05	0.20	% w/w DS
Bulk Density	10	1300	g/l DS
Bulk Density	10	1400	g/l OS
Sieve analysis > 0.063 mm		68.8	% DS
Total sand (0.063 mm -2 mm)	1	62	% w/w ds
Total silt (0.002 mm - 0.063 mm)	1	28	% w/w ds
Clay (< 0.002 mm)	1	10	% w/w ds
Max. water capacity			
pF 1,8		17.7	w %, w ($p_{1.8}$)
pF 1,8		25.9	Vol %, $\Theta(p_{1.8})$
wk max		30.0	w % w(p_m)
wk max		44.0	Vol %, $\Theta(p_m)$
Determination from the aqua regia digestion			
Lead	2	23	mg/Kg DS
Cadmium	0.2	< 0.2	mg/Kg DS
Calcium	20	5400	mg/Kg DS
Total chromium	1	13	mg/Kg DS
Iron	5	16000	mg/Kg DS
Potassium	5	1900	mg/Kg DS
Cobalt	1	8	mg/Kg DS
Copper	1	15	mg/Kg DS
Magnesium	30	3600	mg/Kg DS
Manganese	1	270	mg/Kg DS
Sodium	10	110	mg/Kg DS
Nickel	1	12	mg/Kg DS
Phosphorus	10	461	mg/Kg DS
Zinc	1	63	mg/Kg DS

Supplementary Table S2. Comparison between nominal and analytical concentrations obtained for the active ingredient (glyphosate) and estimated concentration of the adjuvant POEA for Montana® formulation in the soil samples used for the reproduction test of *Folsomia candida*. Limit of quantification (LOQ) was 0.05 mg/kg and recovery rates were within the range of 90-100%.

Sample	Glyphosate Nominal Concentration (mg/kg d.w.)	Glyphosate Analytical Concentration (mg/kg d.w.)	Estimated POEA Concentration (mg/kg d.w.)
Blank	0.00	<0.05	0.00
C1	0.135	0.15	0.028-0.05
C2	0.27	0.19	0.035-0.06
C3	0.54	0.29	0.05-0.09
C4	1.08	0.46	0.08-0.15
C5	2.16	2.40	0.44-0.76
C6	4.32	4.0	0.74-1.26
EC50	4.63	4.71	0.87-1.49

Supplementary Table S3. Mapping sucess and library size of the F. candida transcriptomic data for all samples used.

Sample	Library size	Norm.factors	Mapping_success
M-4d-Ct1	1238042	0,806832478	84,00
M-4d-Ct2	1171209	0,943243745	85,41
M-4d-Ct3	1220710	0,800502384	79,48
M-4d-Ct4	1029405	0,929617621	84,06
M-4d-EC1	1146310	0,763455948	83,67
M-4d-EC2	1206891	0,691542231	85,56
M-4d-EC3	1024094	0,816960811	83,67
M-4d-EC4	1110364	0,735848091	76,53
M-7d-Ct1	720197	0,895576666	85,89
M-7d-Ct2	848792	0,904072057	87,50
M-7d-Ct3	895177	0,883990412	88,08
M-7d-Ct4	1033743	1,015288301	84,40
M-7d-EC1	776787	0,882561163	87,42
M-7d-EC2	832860	1,023217768	85,22
M-7d-EC3	811615	1,030604606	80,56
M-7d-EC4	808539	0,855293585	82,64
M-10d-Ct1	1044374	0,786389535	84,41
M-10d-Ct2	1018250	1,11208786	84,03
M-10d-Ct3	987113	1,085230613	84,84
M-10d-Ct4	1058690	1,124834941	83,83
M-10d-EC1	1092173	1,208645414	73,48
M-10d-EC2	1123560	1,126326195	78,56
M-10d-EC3	956023	1,083366478	83,80
M-10d-EC4	969529	1,15027951	81,27
Average	1005185,29	0,94399	83,26

Supplementary Table S4: Significant differentially expressed transcripts ($p < 0.05$) in *Folsomia candida* after exposure to the herbicide formulation for 4, 7 and 10 days.

Acc Number	Blast Homology	log2 Fold Change	p-value	p-value after FDR
**Transcripts differentially expressed after 4 days of exposure**				
down-regulated				
GAMN01014780	Calcium-activated chloride channel regulator	-5,2332	4,71E-08	0,0018
GAMN01004704	chaperone protein	-3,4533	1,09E-07	0,0021
GAMN01007081	chloride channel calcium activated 2-like	-8,3627	1,84E-07	0,0023
GAMN01010449	fatty acid synthase	-2,4152	1,51E-06	0,0144
GAMN01014598	leucine aminopeptidase	-3,0514	5,31E-06	0,0404
GAMN01007411	Protein of unknown function	-4,4908	6,69E-06	0,0424
GAMN01000321	Protein of unknown function	-2,7572	9,84E-06	0,0474
GAMN01012505	Calcium-activated chloride channel regulator	-7,4635	9,98E-06	0,0474
**Transcripts differentially expressed after 7 days of exposure**				
up-regulated				
GAMN01003982	moxd1 protein	2,4023	7,41E-08	0,0028
GAMN01007179	Protein G12	3,5869	5,19E-07	0,0099
GAMN01013249	-	5,4933	4,50E-06	0,0441
GAMN01005039	glucosylceramidase	2,4141	5,30E-06	0,0441
GAMN01004068	dbh-like monooxygenase protein 2 homolog	2,6813	5,91E-06	0,0441
GAMN01011327	-	3,4368	7,10E-06	0,0441
GAMN01000273	-	2,1910	8,13E-06	0,0441
**Transcripts differentially expressed after 10 days of exposure**				
up-regulated				
GAMN01010546	-	3,3029	6,63E-09	0,0002
GAMN01007291	pupal cuticle protein 78e	2,0872	9,32E-06	0,0091
GAMN01012869	-	1,3850	3,55E-05	0,0217
GAMN01036340	-	7,0278	3,90E-05	0,0235
GAMN01000804	-	4,6449	4,24E-05	0,0248
GAMN01016964	alternative protein dyz1l18	5,7863	9,01E-05	0,0381
down-regulated				
GAMN01006077	Fatty acyl-CoA hydrolase precursor	-7,5224	1,30E-08	0,0002
GAMN01011702	-	-4,4847	3,01E-08	0,0004
GAMN01011300	lipase 3 precursor	-4,5595	5,48E-08	0,0005
GAMN01006678	-	-3,5563	8,73E-08	0,0007
GAMN01003636	-	-3,6806	1,22E-07	0,0008
GAMN01027655	polyhydroxybutyrate depolymerase	-4,5350	2,43E-07	0,0013
GAMN01011789	Sphingomyelin phosphodiesterase	-4,0019	3,32E-07	0,0016
GAMN01004460	polyhydroxybutyrate depolymerase	-3,2426	4,53E-07	0,0018
GAMN01012184	Fatty acyl-CoA hydrolase precursor	-4,2080	4,65E-07	0,0018
GAMN01031326	-	-6,8000	5,36E-07	0,0019
GAMN01001830	-	-3,6197	8,84E-07	0,0028
GAMN01001593	Mucin like protein	-3,5630	1,13E-06	0,0033

GAMN01007221	Endoribonuclease	-2,7394	1,30E-06	0,0033
GAMN01010345	-	-3,2546	1,36E-06	0,0033
GAMN01001426	u11 u12 small nuclear ribonucleoprotein 35 kda	-3,8871	1,41E-06	0,0033
GAMN01011799	pancreatic triacylglycerol lipase-like	-3,2216	1,48E-06	0,0033
GAMN01012686	-	-3,7984	1,56E-06	0,0033
GAMN01006832	Epididymal secretory protein like	-2,4927	1,73E-06	0,0035
GAMN01002516	-	-4,2263	1,98E-06	0,0036
GAMN01008214	-	-3,8925	1,99E-06	0,0036
GAMN01011403	Dopamine beta-hydroxylase	-3,3420	2,47E-06	0,0043
GAMN01001065	Multiple epidermal growth factor-like domain protein 6	-3,0009	2,66E-06	0,0044
GAMN01005688	-	-3,7155	2,78E-06	0,0044
GAMN01019740	collagen alpha-1 chain	-3,0194	4,89E-06	0,0074
GAMN01009595	-	-4,1383	5,32E-06	0,0076
GAMN01008681	-	-3,3460	5,36E-06	0,0076
GAMN01002343	Platelet-activating factor acetylhydrolase IB	-3,0678	5,87E-06	0,0076
GAMN01006374	Zinc carboxypeptidase A 1	-3,0407	6,12E-06	0,0076
GAMN01010918	vitelline membrane outer layer protein 1 homolog	-2,6430	6,34E-06	0,0076
GAMN01013576	Platelet-activating factor acetylhydrolase IB	-3,6530	6,44E-06	0,0076
GAMN01010081	Desert hedgehog protein	-2,2617	6,52E-06	0,0076
GAMN01037556	chitinase partial	-4,0538	6,63E-06	0,0076
GAMN01010400	phb depolymerase	-2,8582	7,96E-06	0,0086
GAMN01026586	Serine--tRNA ligase	-3,3279	8,06E-06	0,0086
GAMN01001119	fibronectin i domain-containing protein	-2,9419	8,14E-06	0,0086
GAMN01027214	penicillin-binding protein	-4,0505	8,47E-06	0,0086
GAMN01008551	Astacin	-2,9278	8,55E-06	0,0086
GAMN01027440	-	-3,0057	1,37E-05	0,0129
GAMN01009798	pancreatic triacylglycerol lipase	-4,4272	1,39E-05	0,0129
GAMN01010659	-	-2,9221	1,47E-05	0,0133
GAMN01001993	vitelline membrane outer layer protein 1 homolog	-2,6779	1,63E-05	0,0145
GAMN01037272	1-acyl-sn-glycerol-3-phosphate acyltransferase beta-like	-3,0687	2,13E-05	0,0178
GAMN01010825	1-acyl-sn-glycerol-3-phosphate acyltransferase beta-like	-3,0687	2,13E-05	0,0178
GAMN01016240	dna rna non-specific endonuclease	-4,1099	2,15E-05	0,0178
GAMN01001848	-	-3,1754	2,28E-05	0,0184
GAMN01005839	collagen alpha-1 chain	-2,5721	2,35E-05	0,0186
GAMN01001649	-	-2,4222	2,43E-05	0,0187
GAMN01037019	Papilin	-4,9322	2,46E-05	0,0187
GAMN01010398	-	-2,5710	2,61E-05	0,0192
GAMN01011116	serine protease family	-2,8896	2,72E-05	0,0192
GAMN01025128	aminopeptidase n	-3,2888	2,72E-05	0,0192
GAMN01009329	-	-3,3838	2,79E-05	0,0192
GAMN01013314	-	-2,7242	2,80E-05	0,0192
GAMN01011814	-	-5,0365	2,83E-05	0,0192
GAMN01000607	-	-2,7924	3,03E-05	0,0202
GAMN01021325	Aggrecan core protein	-4,9140	3,09E-05	0,0202
GAMN01002009	Acetylcholinesterase	-2,6842	3,16E-05	0,0203

GAMN01010702	-	-3,8249	3,32E-05	0,0211
GAMN01010350	Exodeoxyribonuclease 7 large subunit	-2,5260	3,47E-05	0,0216
GAMN01013720	Fatty acyl-CoA hydrolase	-2,7904	4,07E-05	0,0242
GAMN01010274	Proteoglycan	-3,7830	4,37E-05	0,0252
GAMN01001415	leucyl aminopeptidase	-3,1282	4,52E-05	0,0257
GAMN01022313	-	-4,3829	4,84E-05	0,0271
GAMN01001459	n-acetylmuramoyl-l-alanine amidase	-3,0410	5,07E-05	0,0279
GAMN01017940	protein cbd-1	-6,2154	5,21E-05	0,0283
GAMN01010945	Zinc metalloproteinase nas-14	-2,8337	5,42E-05	0,0290
GAMN01000308	-	-2,3486	5,58E-05	0,0291
GAMN01002038	-	-3,1058	5,58E-05	0,0291
GAMN01028231	Beta-lactamase	-4,2705	5,88E-05	0,0295
GAMN01016366	-	-2,5828	5,92E-05	0,0295
GAMN01002969	-	-2,4321	6,05E-05	0,0295
GAMN01008799	dbh-like monooxygenase protein 1	-4,5634	6,09E-05	0,0295
GAMN01001816	fatty acid-binding protein	-2,5754	6,24E-05	0,0295
GAMN01010525	-	-1,9619	6,25E-05	0,0295
GAMN01012003	Peritrophin-1	-3,2782	6,26E-05	0,0295
GAMN01026507	-	-2,7880	6,33E-05	0,0295
GAMN01012819	aminopeptidase n-like protein	-4,3353	6,41E-05	0,0295
GAMN01025562	alkaline tissue-nonspecific isozyme precursor	-5,4563	6,51E-05	0,0295
GAMN01005102	dna rna non-specific endonuclease	-3,1297	6,53E-05	0,0295
GAMN01002719	Copine-D	-3,0867	7,46E-05	0,0334
GAMN01010521	-	-2,3216	7,96E-05	0,0350
GAMN01013655	Cytochrome protein	-3,8160	8,09E-05	0,0350
GAMN01011019	-	-2,9586	8,11E-05	0,0350
GAMN01005786	-	-3,6408	8,91E-05	0,0380
GAMN01008482	sphingomyelin phosphodiesterase	-3,4617	9,11E-05	0,0381
GAMN01001845	carboxypeptidase a-like	-2,7271	9,67E-05	0,0400
GAMN01001159	-	-3,0326	9,79E-05	0,0400
GAMN01011743	alkaline phosphatase	-2,7778	1,00E-04	0,0405
GAMN01003952	-	-2,5680	1,06E-04	0,0422
GAMN01029760	membrane protein	-4,6349	1,08E-04	0,0428
GAMN01007170	chitinase	-2,4909	1,17E-04	0,0456
GAMN01027507	Peritrophin-1	-1,9602	1,17E-04	0,0456
GAMN01012849	leucine aminopeptidase	-3,2269	1,19E-04	0,0458
GAMN01006525	-	-2,6990	1,24E-04	0,0471
GAMN01037991	-	-2,5002	1,27E-04	0,0478
GAMN01011217	Proteoglycan like	-2,5647	1,34E-04	0,0499

Supplementary Table S5: All significant down- and up-regulated transcripts of the significant (p<0.05) gene ontology (GO) terms (Biological Process and Molecular Function) in Folsomia candida after exposure to Montana® herbicide for 4, 7 and 10 days.

GO ID	Domain	Term	Library Annotated	# Sig. Annotated	p-Value	Significant Transcripts (Accession number)
Transcripts differentially expressed after 4 days of exposure						
down-regulated						
GO:0006633	BP	fatty acid biosynthetic process	60	1	0,019	GAMN01010449
GO:0004313	MF	[acyl-carrier-protein] S-acetyltransferase activity	10	1	0,0026	GAMN01010449
GO:0004316	MF	3-oxoacyl-[acyl-carrier-protein] reductase (NADPH) activity	10	1	0,0026	GAMN01010449
GO:0004317	MF	3-hydroxypalmitoyl-[acyl-carrier-protein] dehydratase activity	10	1	0,0026	GAMN01010449
GO:0004319	MF	enoyl-[acyl-carrier-protein] reductase (NADPH, B-specific) activity	10	1	0,0026	GAMN01010449
GO:0004320	MF	oleoyl-[acyl-carrier-protein] hydrolase activity	10	1	0,0026	GAMN01010449
GO:0016295	MF	myristoyl-[acyl-carrier-protein] hydrolase activity	10	1	0,0026	GAMN01010449
GO:0016296	MF	palmitoyl-[acyl-carrier-protein] hydrolase activity	10	1	0,0026	GAMN01010449
GO:0004314	MF	[acyl-carrier-protein] S-malonyltransferase activity	11	1	0,0029	GAMN01010449
GO:0004315	MF	3-oxoacyl-[acyl-carrier-protein] synthase activity	11	1	0,0029	GAMN01010449
GO:0031177	MF	phosphopantetheine binding	12	1	0,0031	GAMN01010449
GO:0004177	MF	aminopeptidase activity	36	1	0,0093	GAMN01014598
Transcripts differentially expressed after 7 days of exposure						
up-regulated						
GO:0046519	BP	sphingoid metabolic process	13	1	0,0042	GAMN01005039
GO:0055114	BP	oxidation-reduction process	1100	2	0,0391	GAMN01003982; GAMN01004068
GO:0004348	MF	glucosylceramidase activity	12	1	0,0031	GAMN01005039
GO:0016491	MF	oxidoreductase activity	1271	2	0,0337	GAMN01003982; GAMN01004068
Transcripts differentially expressed after 10 days of exposure						
up-regulated						
GO:0005214	MF	structural constituent of chitin-based cuticule	27	1	0,0023	GAMN01007291
down-regulated						
GO:0006030	BP	chitin metabolic process	155	4	6,90E-06	GAMN01010400; GAMN01009329; AMN01000607; GAMN01012003
GO:0006508	BP	proteolysis	827	11	2,10E-05	GAMN01008551; GAMN01006374; GAMN01037019; GAMN01025128; GAMN01001845; GAMN01001415; GAMN01012819; GAMN01012849; GAMN01010945; GAMN01011116; GAMN01010081
GO:0030704	BP	vitelline membrane formation	19	2	0,0016	GAMN01019821; GAMN01010918
GO:0006685	BP	sphingomyelin catabolic process	2	1	0,0063	GAMN01008482
GO:0008654	BP	phospholipid biosynthetic process	73	2	0,0217	GAMN01010825; GAMN01037272
GO:0009253	BP	peptidoglycan catabolic process	8	1	0,0248	GAMN01001459
GO:0008061	MF	chitin binding	135	5	2,70E-05	GAMN01010400; GAMN01012003; GAMN01037556; GAMN01000607; GAMN01009329

GO:0003841	MF	1-acylglycerol-3-phosphate O-acyltransferase activity	6	2	0,0001	GAMN01010825; GAMN01037272
GO:0016787	MF	hydrolase activity	2751	5	0,00018	GAMN01013576; GAMN01016240; GAMN01002343; GAMN01025128; GAMN01005102
GO:0004568	MF	chitinase activity	27	2	0,00235	GAMN01037556; GAMN01007170
GO:0004181	MF	metallocarboxypeptidase activity	30	2	0,0029	GAMN01018349; GAMN01006374

Supplementary Table S6: Detected protein levels in *Folsomia candida* after exposure to Montana® herbicide for 4, 7 and 10 days.

Transcript Acc. number	Protein homolog	Unused score	Fold Change (Log2)	p-value
Protein levels after 4 days of exposure				
up-regulated				
GAMN01010129	Protein of unknown function	18	0,0315	0,9723
GAMN01001955	ATP synthase subunit beta, mitochondrial	8,02	0,7878	0,2000
GAMN01010555	50S ribosomal protein L9	6	0,4548	0,5445
GAMN01009525	Actin, muscle	6	0,1106	0,6876
GAMN01003161	Tropomyosin	4	0,3383	0,5656
GAMN01001328	Alpha-actinin, sarcomeric	4	0,7796	0,2181
GAMN01006975	60 kDa heat shock protein, mitochondrial	4	0,8326	0,0848
GAMN01013656	Myosin heavy chain, muscle	2	0,0616	0,8953
GAMN01006965	Myosin heavy chain, muscle	2	0,3746	0,4715
GAMN01007545	Myosin heavy chain, muscle	2	0,4244	0,5227
GAMN01011098	Arginine kinase	2	5,6153	0,0798
GAMN01001293	Calexcitin-2	2	0,8764	0,0922
GAMN01012020	Eukaryotic translation initiation factor 2A	2	0,0012	0,9977
GAMN01003618	Bacillopeptidase F	2	-	-
GAMN01000520	Cofilin/actin-depolymerizing factor	2	0,1757	0,7545
GAMN01000325	40S ribosomal protein S19	2	0,0852	0,4634
GAMN01001650	Elongation factor 2	2	0,2168	0,6132
down-regulated				
GAMN01008776	Vitellogenin-6	4	-0,0214	0,9601
GAMN01026524	Larval Cuticle protein	3,05	-0,3316	0,9121
GAMN01000415	Glutathione S-transferase Mu 1	2	-0,2727	0,8273
GAMN01001896	Protein of unknown function	2	-0,5145	0,3895
Protein levels after 7 days of exposure				
up-regulated				
GAMN01003161	Tropomyosin	14,44	0,1397	0,7961
GAMN01003691	Actin	10	0,4939	0,3314
GAMN01006818	Vitellogenin-2	9,09	1,4673	0,0060
GAMN01009208	Vitellogenin	8,07	2,7101	0,0011
GAMN01014973	Endocuticle structural glycoprotein SgAbd-2	4	0,1811	0,5711
GAMN01010555	50S ribosomal protein L9	2,12	3,7592	0,0049
GAMN01001293	Calexcitin-2	2,11	0,0377	0,6749
GAMN01000775	Histone H3	2,09	0,0183	0,9099
GAMN01002103	ADP,ATP carrier protein	2	1,5475	0,0075
GAMN01032080	Cytochrome c	2	3,3266	0,0001
GAMN01003618	Bacillopeptidase F	2	0,4098	0,4824
GAMN01014014	cuticle protein-like	2	0,7478	0,0863
GAMN01024903	Protein of unknown function	2	0,0751	0,4682
GAMN01000325	40S ribosomal protein S19	2	1,8399	0,0081
GAMN01009769	Troponin C, isoform 2	2	0,6128	0,2174
GAMN01007158	Peptidyl-prolyl cis-trans isomerase	2	0,0973	0,9036

GAMN01007174	40S ribosomal protein S4	1,43	0,0823	0,8997
GAMN01006965	Myosin heavy chain, muscle	0,87	0,1746	0,6237
GAMN01003635	Heat shock 70 kDa protein cognate 2	0,62	1,7905	0,0178
GAMN01011035	Malate dehydrogenase, mitochondrial	0,14	0,0307	0,8930
GAMN01021109	Protein of unknown function	0,08	0,5747	0,3720
GAMN01007895	Kinesin-related protein	0,06	0,3006	0,2310
GAMN01005939	Protein of unknown function	0,05	0,0575	0,7910
GAMN01002674	Putative ubiquitin -like protein	0,05	0,9234	0,7210
down-regulated				
GAMN01001841	Protein of unknown function	18,12	-0,1357	0,8314
GAMN01001955	ATP synthase subunit beta, mitochondrial	11,62	-0,2090	0,6645
GAMN01006818	Vitellogenin-6	8	-0,0836	0,8438
GAMN01011098	Arginine kinase	4,07	-0,1827	0,0602
GAMN01026524	Cuticle protein	4	-0,1811	0,7293
GAMN01027458	Histone H4	4	-0,1126	0,7301
GAMN01016941	Cuticle protein 7	2	-1,0582	0,1917
GAMN01022936	Cuticle protein 7	2	-0,0189	0,9162
GAMN01015530	Protein of unknown function	2	-0,2966	0,2568
GAMN01009199	Cuticle protein 6	2	-0,6413	0,0290
GAMN01000369	40S ribosomal protein S18	0,09	-0,2696	0,3420
GAMN01002298	Chitooligosaccharidolytic beta-N-acetylglucosaminidase	0,07	-0,0060	0,9640
Protein levels after 10 days of exposure				
up-regulated				
GAMN01004016	Larval cuticle protein A2B	17,98	0,0338	0,9501
GAMN01006818	Vitellogenin-2	14,68	3,9662	0,0166
GAMN01009208	Vitellogenin	14,63	3,0342	0,0220
GAMN01006965	Myosin heavy chain, muscle	12,03	0,0965	0,7494
GAMN01001955	ATP synthase subunit beta, mitochondrial	11,04	0,0617	0,9255
GAMN01001922	Phenoloxidase 3	6	1,1265	0,0904
GAMN01006965	Myosin heavy chain, muscle	4,56	1,3457	0,0533
GAMN01000430	Fatty acid-binding protein, brain	3,77	0,5905	0,0033
GAMN01001328	Alpha-actinin, sarcomeric	2,56	1,4596	0,0023
GAMN01011035	Malate dehydrogenase, mitochondrial	2,01	0,2378	0,5229
GAMN01007126	Heat shock protein 83	2	0,2769	0,4973
GAMN01011769	Troponin T	2	0,0713	0,5537
GAMN01007174	40S ribosomal protein S4	2	0,0084	0,9789
GAMN01010159	Tubulin beta-3 chain	2	0,5476	0,3140
GAMN01012094	Myosin regulatory light chain 2	2	0,6461	0,5170
GAMN01007002	ATP synthase subunit d, mitochondrial	2	0,1316	0,3602
GAMN01010569	ATP synthase subunit alpha, mitochondrial	2	-	-
GAMN01000323	Superoxide dismutase [Cu-Zn]	2	6,3326	2,02E-06
GAMN01010129	Protein of unknown function	0,55	-	-
down-regulated				
GAMN01003161	Tropomyosin	14,35	-0,9920	0,0278
GAMN01003691	Actin	12,1	-0,5049	0,4360
GAMN01011098	Arginine kinase	8	-1,0167	0,1022
GAMN01027458	Histone H4	6	-0,4448	0,5043
GAMN01008776	Vitellogenin-6	6	-0,0088	0,9274
GAMN01026524	Cuticle protein	4	-0,2471	0,5301

GAMN01012020	Eukaryotic translation initiation factor 2A	4	-0,0574	0,6511
GAMN01011769	Troponin T	4	-0,0798	0,7494
GAMN01022936	Cuticle Protein 7	4	-0,2283	0,6759
GAMN01001293	Calexcitin-2	2,01	-0,3653	0,4186
GAMN01001746	Calcium-transporting ATPase sarcoplasmic/endoplasmic reticulum type	2	-0,1281	0,3846
GAMN01026212	Ultrabiothorax	2	-0,5271	0,1070
GAMN01006823	Glutathione S-transferase	2	-0,5912	0,0483
GAMN01001827	Catalase	2	-0,7041	0,2472
GAMN01011144	Endocuticle structural glycoprotein SgAbd-2	2	-0,9817	0,7130
GAMN01007191	ATPase inhibitor, mitochondrial	2	-0,3024	0,1156
GAMN01010257	Tubulin alpha-1 chain	2	-0,1579	0,3645
GAMN01009769	Troponin C, isoform 1	2	-0,4654	0,0932
GAMN01009199	Cuticle protein 6	2	-0,1851	0,7499
GAMN01001896	Protein of unknown function	2	-0,2798	0,0180
GAMN01007158	Peptidyl-prolyl cis-trans isomerase	2	-0,0636	0,8810
GAMN01004453	NADH dehydrogenase [ubiquinone] flavoprotein 2, mitochondrial	2	-0,6392	0,0142
GAMN01015530	Protein of unknown function	1,77	-0,3061	0,0585
GAMN01000369	40S ribosomal protein S18	1,54	-0,0854	0,4521
GAMN01003618	Bacillopeptidase F	0,29	-0,3413	0,3502

Supplementary Table S6: Pearson correlation coefficients and p-values between gene expression and protein levels of different time points (4, 7 and 10 days of exposure). GE (Gene expression) and PL (Protein Levels).

	**All Genes and Proteins**			**Timepoints**			**Stress Related**			**Motor Activity**			**Development**		
	r_p	**p-value**	***N***	r_p	**p-value**	***N***	r_p	**p-value**	***N***	r_p	**p-value**	***N***	r_p	**p-value**	***N***
	-0,13	0,2	99	–	–	–	–	–	–	–	–	–	–	–	–
**4 Days GE to 4 Days PL**	–	–	–	0,148	0,534	20	0,828	0,172	4	**0,998**	**0,011**	4	-0,523	0,366	5
**4 Days GE to 7 Days PL**	–	–	–	**-0,327**	**0,05**	36	-0,531	0,278	6	**0,997**	**0,047**	3	-0,233	0,546	9
**4 Days GE to 10 Days PL**	–	–	–	-0,152	0,33	43	**0,595**	**0,007**	10	0,057	0,903	7	0,233	0,536	10
**7 Days GE to 7 Days PL**	–	–	–	**-0,395**	**0,017**	36	-0,48	0,336	6	0,6	0,591	3	0,078	0,842	9
**7 Days GE to 10 Days PL**	–	–	–	**-0,443**	**0,003**	43	**-0,661**	**0,037**	10	-0,351	0,44	7	0,159	0,661	10
**10 Days GE to 10 Days PL**	–	–	–	-0,037	0,81	43	**0,84**	**0,002**	10	0,56	0,191	7	0,263	0,463	10

Supplementary Figure S1: Heat plot of transcript expression clustering using the SOTA algorithm and individual clusters with the representative differentially expressed genes for each cluster after *Folsomia candida* exposure to Montana® herbicide (4.71 mg glyphosate/kg soil) during 4, 7 and 10 days.

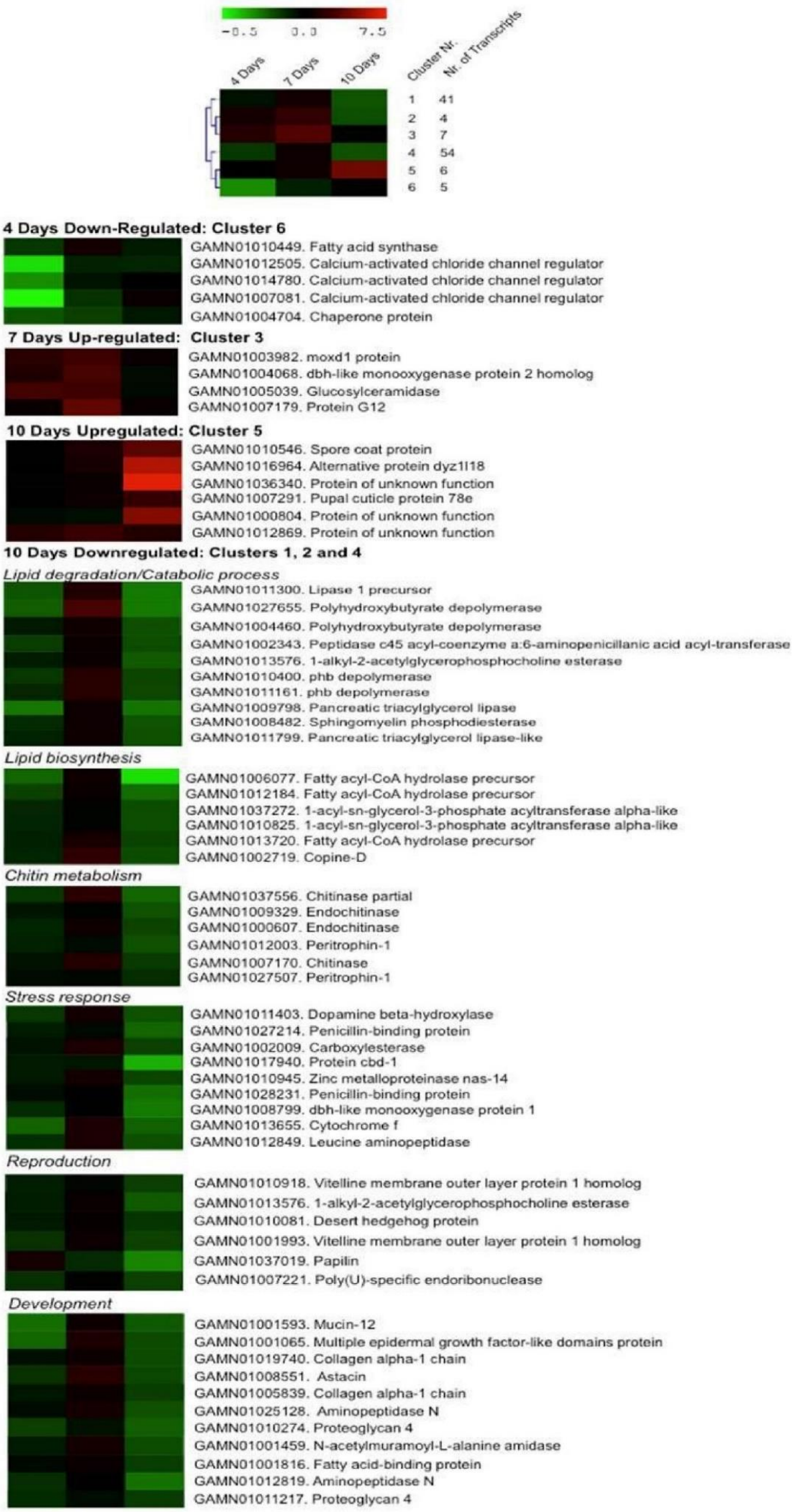

Supplementary Figure S2: KEGG pathway analysis performed with *Folsomia candida*'s differentially expressed transcripts after exposure to Montana® herbicide, using KEGG database online (Kanehisa *et al*, 2017)[52]. Lipid or lipid related metabolic pathways affected are highlighted in red.

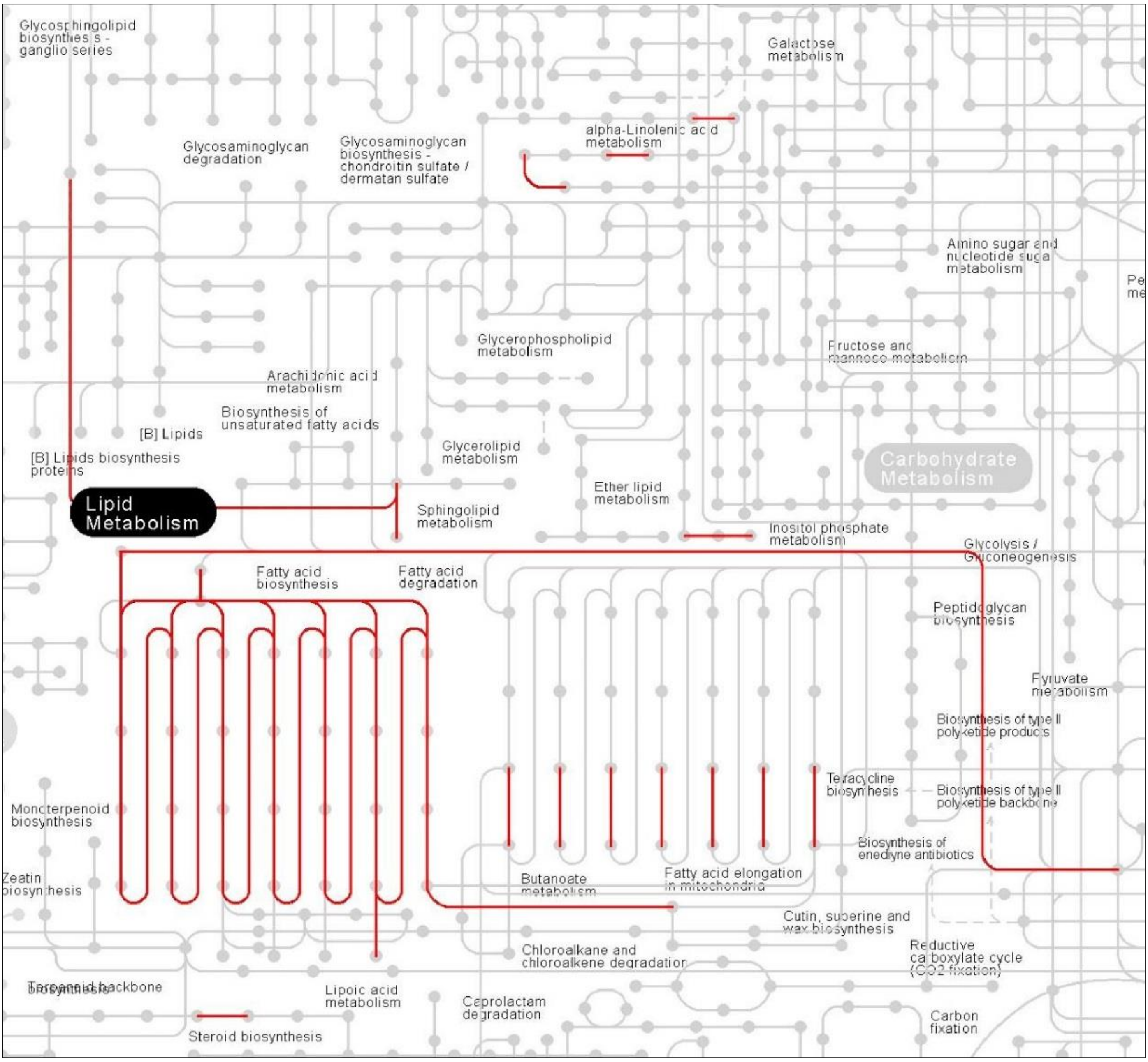

Supplementary Text. Supplementary methodological information on fractionation conditions for the peptide separation (2D).

1. First Peptide Fractionation (pH=10)

For the first fractionation, an Ettan LC chromatograph (GE Healthcare, Diegem, Belgium) was used, equipped with a 50 µL loop and a Luna 5u C18 column (150×2.0 mm, 5 µm, 100 Å; Phenomenex, CA, US). The LC pump was operating at a flow rate of 0.05 mL/min. The mobile phases were 20mM ammonium formate, pH 10, in 2% acetonitrile (ACN) as solvent A, and 20mM ammonium formate in 80% ACN as solvent B. About 100 µg of combined labelled samples resuspended in 50 µL solvent A were used for injection. The separation gradient consisted of a linear increase from 0% to 50% solvent B over 30 min, followed by 50% to 100% solvent B over 1 min. This percentage was maintained for 5 min prior to returning to the starting conditions. The eluted peptides from the first fractionation were collected every 1 min in different wells of 96-well plates. The absorbance of the effluent was registered at 214, 220, and 280 nm by a UV-900 monitor (GE Healthcare, UK).

2. Second Peptide Fractionation (pH=3)

Samples were collected by a Famos microautosampler (Dionex-LC Packings, Hercules, CA, US) onto a RP trapping column (Pepmap C18, 800 µm×5 mm) using the Switchos device, equipped with two micro 2-position/10 port valves (Dionex-LC Packings). The loading pump was set to 10 µL/min, using 0.05% TFA in 5% ACN as the mobile phase. After 8 min, the valve was switched and the sample was eluted onto the analytical separation column (Pepmap C18, 75 µm×150 mm), in a back flush mode, using the nano-LC pump operating at 0.3 µL/min. At this point, the mobile phases were 0.1% TFA in 5% ACN (solvent A) and 0.1% TFA in 80% ACN (solvent B). Peptides were resolved by gradient elution with a linear increase from 0% to 50% solvent B over 30 min, followed by 50% to 100% solvent B over 10 min. This was maintained for another 5 min before returning to the initial conditions. The column effluent was monitored at 214 nm using a 3 nL UV flow cell (Dionex-LC 720 Packings).
